# Urological Manifestations of Kindler Syndrome: A Case Report

**DOI:** 10.7759/cureus.24758

**Published:** 2022-05-05

**Authors:** Rudra Ghorai, Gurpremjit Singh, Ankur Mittal, Vikas K Panwar, Harkirat Talwar

**Affiliations:** 1 Urology, All India Institute of Medical Sciences, Rishikesh, IND

**Keywords:** kindler syndrome, urological manifestation of kindler syndrome, genetic counseling, fermt1, meatal stenosis

## Abstract

Kindler syndrome is a rare autosomal recessive skin disorder. It results from mutation of the FERM domain containing kindlin-1 (FERMT1) that leads to loss of function of kindlin-1, which plays a role in keratinocyte adhesion, polarization, proliferation, and migration. It is characterized by skin blistering, photosensitivity, progressive poikiloderma, and skin atrophy. The mucosae genitourinary system is commonly affected. The urological manifestations include meatal stenosis, urethral stricture, phimosis, and scarring of the glans penis. Skin biopsy with genetic analysis is the gold standard for diagnosis. Genetic counseling and a multidisciplinary approach are the mainstays of treatment.

## Introduction

Kindler syndrome is an autosomal recessive, rare cutaneous disease of hereditary epidermolysis bullosa and poikiloderma congenitale [1,2]. Theresa Kindler first described it in 1954 [3]. Kindler syndrome is characterized by skin fragility with trauma-induced blistering, skin atrophy, photosensitivity, poikiloderma, hyperkeratosis of palms and soles, pseudo syndactyly, mucosal involvement of eyes, mouth, periodontium, gastrointestinal tract, vagina, and urethra. It results from mutation of the FERM domain containing kindlin-1 (FERMT1) that leads to loss of function of kindlin-1, which plays a role in keratinocyte adhesion, polarization, proliferation, and migration. Loss of function of kindlin-1 causes the skin to be fragile and form a blister. Skin atrophy occurs due to defects in actin-extracellular matrix linkage [2,4]. Approximately 250 cases of Kindler's syndrome have been reported worldwide. Urological manifestations of Kindler syndrome include meatal urethral stenosis, urethral stricture, phimosis, and scarring of the glans penis [5]. Here we report a case of Kindler syndrome attending the urology outpatient department.

## Case presentation

**Figure 1:**
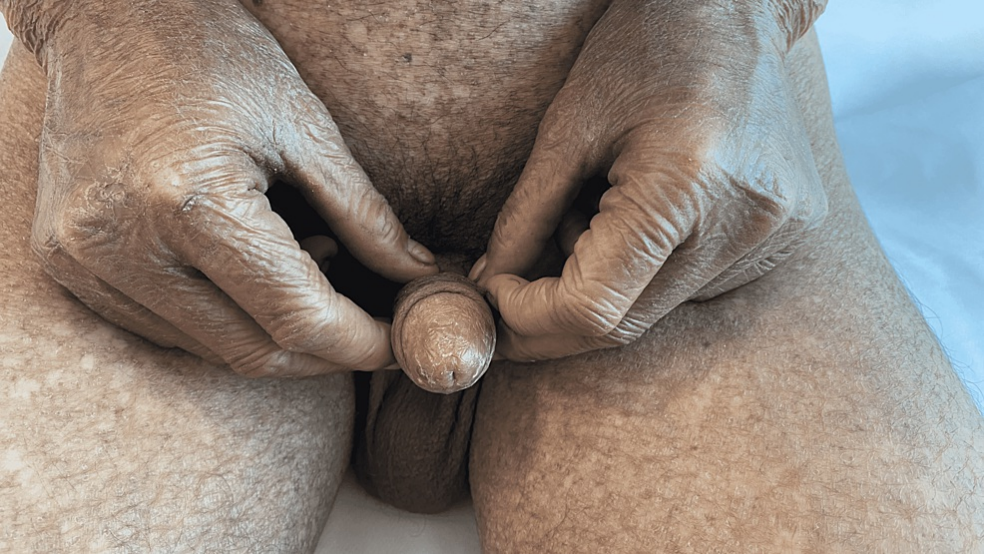
Skin manifestations of Kindler syndrome showing hypopigmented and hyperpigmented patches, hyperkeratosis, cigarette paper-like wrinkling on the dorsum of hands

A 17-year-old male presents with complaints of voiding lower urinary tract symptoms and blackish to brownish color pigmentation all over the body, starting over the neck and face and then involving the whole body. The patient had a history of photosensitivity with the episode of gum bleeding, blistering over the upper and lower limbs since birth, and blistering and erosion over the oral and genital mucosa. His two siblings also have similar complaints with no history of consanguineous marriage. On clinical examination, he had extensive poikilodermatous changes with atrophy, reticulate pigmentation, and telangiectasia all over the body. The dorsum of his hands had characteristics of "cigarette paper" wrinkling with dystrophic nails (Figure 1).

**Figure 2:**
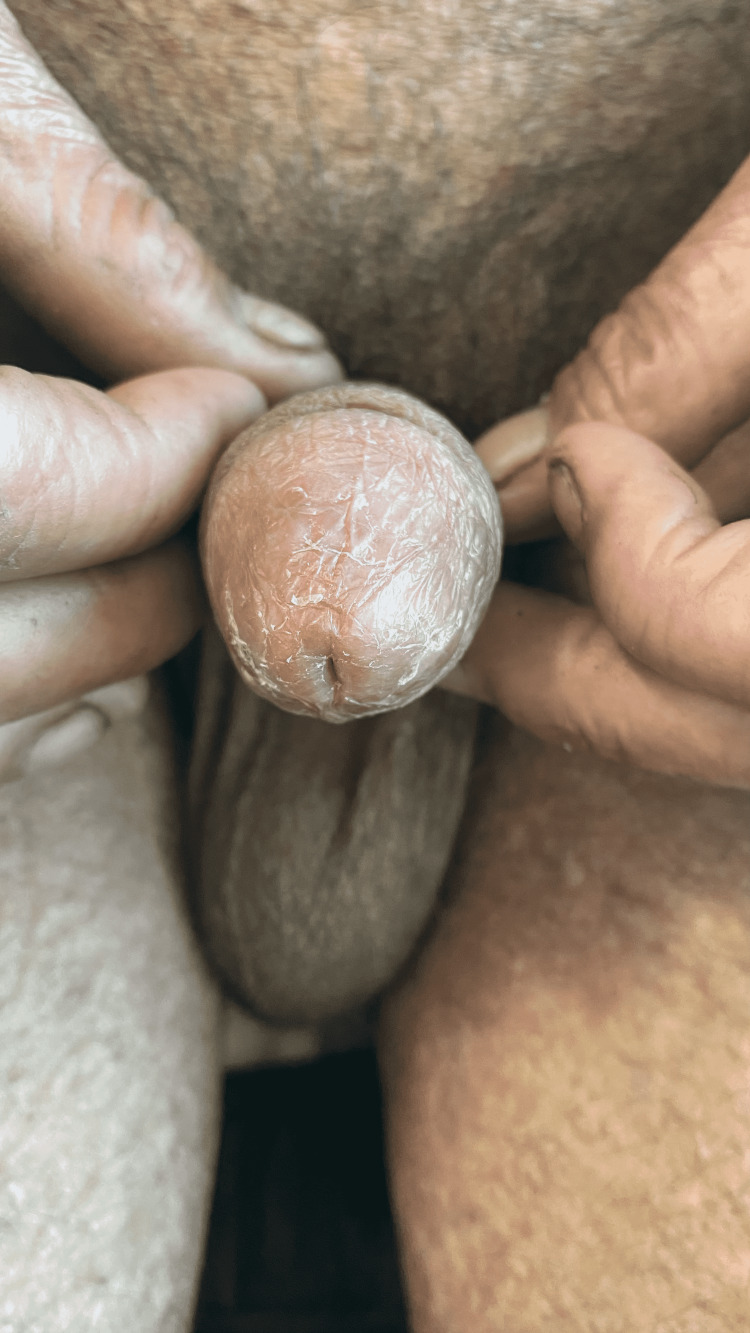
Meatal stenosis with scarring of the glans penis

Genital examination showed meatal stenosis with scarring over the glans (Figure 2).

**Figure 3:**
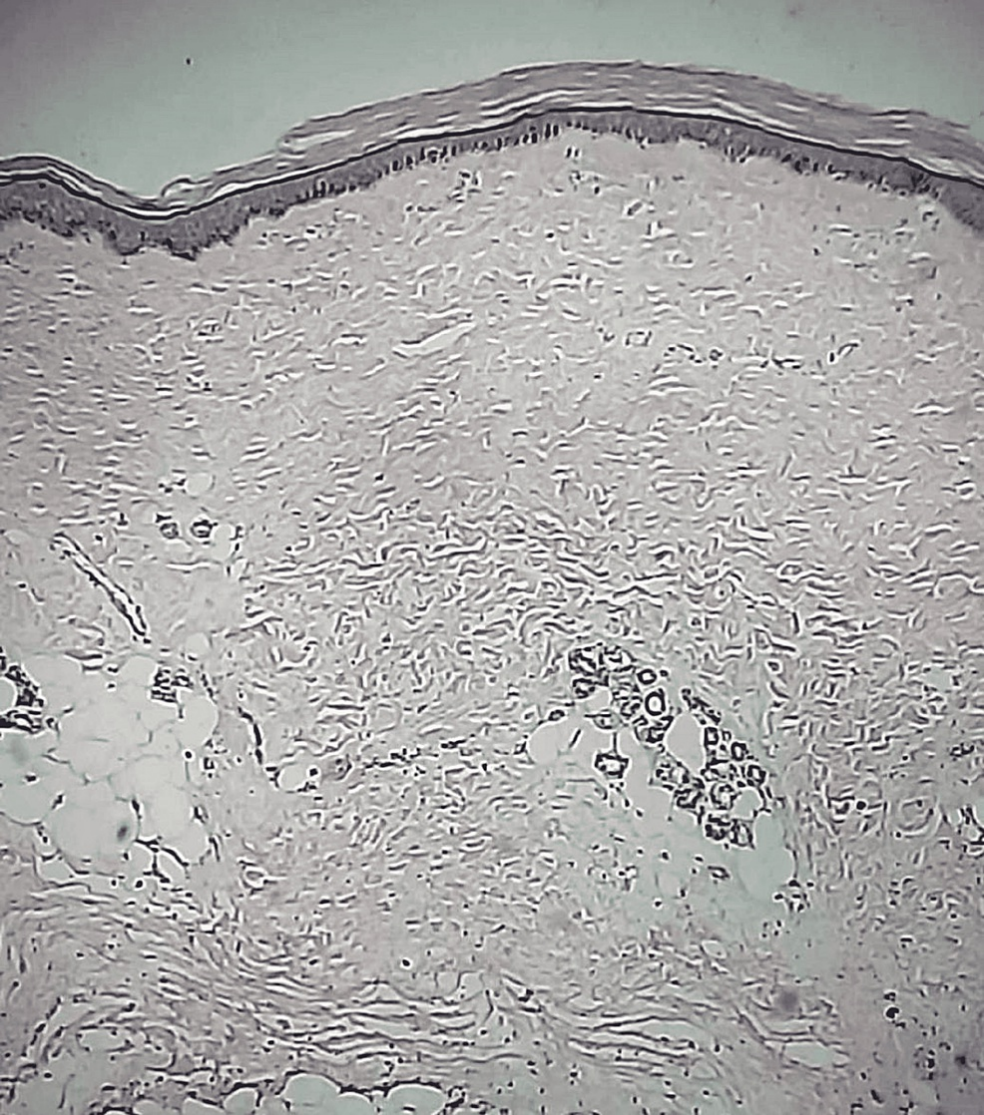
Biopsy of skin showed subepidermal acantholysis and telangiectasia

Systemic examination and laboratory investigations were within normal limits. Uroflowmetry was suggestive of voiding with abdominal straining. A biopsy of the skin showed subepidermal acantholysis and telangiectasia (Figure 3).

Genetic analysis could not be done due to a lack of resources. The patient underwent meatal dilatation, and subsequently, he was relieved of symptoms.

## Discussion

Kindler syndrome is a type of epidermolysis bullosa. Mutation of FERMT1 leads to defective kindlin-1, resulting in blistering at multiple cleavage planes like intraepidermal, junctional, and sublamina densa. Differentiating points of Kindler syndrome with other types of epidermolysis bullosa are exquisite to photosensitivity and poikiloderma [6].

Although cutaneous manifestations improve with age, systemic complications occur with advanced age, such as mucosal ulceration and stenosis leading to esophageal stricture, anal stenosis, urogenital strictures, scarring of conjunctiva, corneal erosion, and ectropion of eyelids and increase the risk of nonmelanoma skin cancer. There are reported cases of squamous cell cancer of the lower lip, transitional cell cancer of the bladder, and laryngeal squamous cell cancer [7]. Differential diagnoses of Kindler syndrome are Weary syndrome, Rothmund Thompson syndrome, Bloom syndrome, and Cockayne syndrome [3].

In the absence of genetic studies, the diagnosis of Kindler syndrome depends on the proposed criteria of Fischer et al. [8]. Here, in our case, the presence of all four major criteria confirms the diagnosis [9]. No established treatment is available for Kindler syndrome. Treatment is symptomatic and preventive, like preventing skin trauma, heat exposure, wound care, good nutritional support, surveillance for extracutaneous manifestations, and psychosocial support with genetic counseling [10]. Urinary tract instrumentation should be judicious to avoid trauma and subsequent stricture formation. If required, small-sized instruments should be used [5,11]. A multidisciplinary team of dermatologists, ophthalmologists, dentists, urologists, gastroenterologists, pediatricians, nurse specialists, dieticians, psychologists, and geneticists is required to treat Kindler syndrome patients.

## Conclusions

In a patient with meatal stenosis with scarring of penile glans and associated cutaneous abnormalities, Kindler syndrome should be considered as a differential diagnosis, and systemic evaluation should be done to assess other associations of this syndrome. Prevention of skin trauma, avoidance of heat exposure, proper wound care, psychosocial support with genetic counseling, and multidisciplinary treatment for symptomatic relief is needed for the treatment of such patients. 
